# Determination of Gender from Various Measurements of the Humerus

**DOI:** 10.7759/cureus.6598

**Published:** 2020-01-08

**Authors:** Muhammad Amjad Khan, Humara Gul, Shahid Mansor Nizami

**Affiliations:** 1 Anatomy, Bakhtawar Amin Medical and Dental College, Multan, PAK; 2 Anatomy, Abwa Medical College, Faisalabad, PAK; 3 Anatomy, Nishtar Medical University, Multan, PAK

**Keywords:** gender, determine, humerus, forensics, skeletal remains

## Abstract

Objective

To study different measurements of the humerus for the determination of sex

Study design

A cross-sectional study

Place and duration of study

The Anatomy Department of Nishtar Medical University and Hospital from June 1, 2018, to May 31, 2019.

Methodology

Six measurements, including maximum length of the humerus (MLH), maximum diameter of the head of the humerus (MDH), vertical diameter of the head of the humerus (VDH), transverse diameter of the head of the humerus (TDH), epicondylar breadth (EB), and condylar breadth (CB) were calculated for 122 male humeri and 52 female humeri. These variables were compared between both genders using the student’s t-test. Wilks’ lambda test was applied. The demarking point of all these variables was defined as the average of the means of male and female measurements. Correctly identified cases were calculated in the male group, the female group, and the whole study group. Data analysis was done in SPSS v.23.0 (IBM Corp, Armonk, NY). P>0.05 was statistically insignificant.

Results

The differences of MLH, MDH, VDH, TDH, EB, and CB were statistically significant between both the genders (p<0.001). The accuracy of MLH was 85% in the total population. The accuracy of MDH, VDH, and TDH was 76%, 85%, and 76% in the total population, respectively. EB and CB correctly identified 75% and 78% of all the bones.

Conclusion

This study shows that maximum humeral length and the vertical diameter of the humeral head are the two most important measurements with the highest accuracy for the determination of gender from the skeletal remains of the human body in the South-East Asian population.

## Introduction

In forensic anthropology, it is very important to determine the sex from the bony remains found at an excavation site or a crime scene. The humerus is a big bone in the upper limb and its condition does not usually deteriorate, which is why the humerus is favored for the determination of sex. The length of various bones in the human body, including the humerus, is a good predictor of gender but the accuracy of the vertical diameter of the head is also significant in sex determination [1-2]. Numerous studies have been conducted on the measurements of upper limb bones including the humerus and metric systems devised in Chile [2], South Africa [3], Guatemala [4], the Dart collection [4-5], the island of Crete [1,6-7], Turkey [8], Greece [9], America [10], and the Eastern Adriatic coast [11].

Many factors are important for forensic specialists for the identification of unknown dead bodies. Estimation of body stature and determination of sex are the most important factors used to establish the identity of unidentifiable dead bodies. Determination of sex is considered an easy job, as external and internal genitalia directly point towards the gender of the individual [12]. But this task becomes a challenge when it comes to severely decomposed dead bodies. Determination of sex is also important in the evaluation of other parameters such as body stature [13]. The simplest method of sex determination is the visual assessment of the bone and the inspection of the bony landmarks distinctive of gender. This qualitative method is more accurate if a significant part of the skeleton is available. Another method used is the morphometric technique and is considered more accurate. This metric method depends on direct measurements between various bony points and the intricate outlines of the bones cannot be evaluated [14-15].

Upper limb bones vary in size among different populations. The length of the humerus is different in the African, American, and European populations. There are significant differences among Asian populations. The discriminant value for the length of the humerus has been calculated for various Asian populations, including Thai, Japanese, and Chinese. Sex determination has been done using the skull, mandible, pelvic bone, hyoid bone [16], corpus callosum [17], and thyroid cartilage in the Korean population [18].

Despite having low accuracy, a determination of sex can be made by the quantitative analysis of the complete bone or even fragments of bones. Although upper limb bones, including the humerus, preserve their integrity, they are usually fragmented at the time of retrieval. This study is directed to derive demarking values for the whole humerus or fragments of the humerus in the south Asian population. The aim of this study is to devise accurate anthropometric standards to determine the sex from different measurements of the humerus. As in our region, this subject remained of least priority; this study will help fill this gap and open new gates of research on this topic.

## Materials and methods

This cross-sectional study was conducted on 174 humeri, which included 122 humeri from male cadavers and 52 humeri from female cadavers. The duration of the study was one year, i.e. from June 1, 2018, to May 31, 2019. The sample size was calculated [19] and the nonprobability consecutive sampling technique was used to select the bones for the study. The study was approved by the review board of Nishtar Medical University and Hospital. All the humeri were removed from the cadavers of adult individuals of estimated age between 25 years and 75 years in the anatomy department. All the bones were examined for any obvious deformity. Bones indicating obvious deformities, including healed fractures, neoplastic bone disease, and developmental defects, were excluded.

All the bones were cleaned after removal from the cadavers. A surgical knife was used to remove the articulate cartilages. An osteometric table, measuring tape, and digital calipers were the instruments of choice for various measurements of the humeri. Six humerus measurements were included, i.e. maximum length of the humerus (MLH), maximum diameter of the head of the humerus (MDH), vertical diameter of the head of the humerus (VDH), transverse diameter of the head of the humerus (TDH), condylar breadth (CB), and epicondylar breadth (EB). The maximum humerus length was defined as the displacement between the top-most projection of the humeral head to the lowest point on the trochlea. VDH was the displacement between the highest and lowest points on the margin of the articular surface of the humeral head. The transverse diameter of the head was the displacement between the most anterior and most posterior points on the margin of the articular surface of the humeral head. Epicondylar breadth was the distance of the two most laterally projecting points on the lateral epicondyles of the humerus.

All these six variables, MLH, MDH, VDH, TDH, EB, and CB, were compared between both genders using the student’s t-test. Wilks’ lambda test was applied for all these measurements. The demarking point of all these variables was defined as the average of the means of male and female measurements. Correctly identified cases were calculated in the male group, female group, and the whole study group. Data analysis was done in SPSS v.23.0 (IBM Corp, Armonk, NY). P>0.05 was statistically insignificant.

## Results

The mean maximum length of the humerus was 304.56 ± 14.16 mm and 276.60 ± 10.89 mm in males and females, respectively (p<0.001). The maximum diameter, vertical diameter, and transverse diameter of the humeral head were 44.95 ± 1.72 mm, 44.73 ± 1.70 mm, and 40.92 ± 1.71 mm in the males while 41.34 ± 2.07 mm, 40.98 ± 1.72 mm, and 38.36 ± 1.69 mm in the females, respectively (p<0.001). Epicondylar breadth was 59.44 ± 3.20 mm in the males and 54.52 ± 2.30 mm in the females (p<0.001). Condylar breadth was 41.23 ± 1.91 mm and 38.73 ± 1.76 mm in the males and females, respectively (p<0.001). See Table 1.

**Table 1:** Variable comparison between two genders

Variable	Male (n=122)	Female (n=52)	p-value
MLH, mm	304.56 ± 14.16	276.60 ± 10.89	<0.001
MDH, mm	44.95 ± 1.72	41.34 ± 2.07	<0.001
VDH, mm	44.73 ± 1.70	40.98 ± 1.72	<0.001
TDH, mm	40.92 ± 1.71	38.36 ± 1.69	<0.001
EB, mm	59.44 ± 3.20	54.52 ± 2.30	<0.001
CB, mm	41.23 ± 1.91	38.73 ± 1.76	<0.001

Table 2 represents the discriminant function coefficient of all the bony measurements separating males from females. A measured value less than the demarking point was labeled as female and a measured value greater than the demarking point was labeled as male. For example, a value above 290.5 mm for the maximum length of the humerus will be considered male whereas a value less than 290.5 mm will be considered female. The demarking points for MDH, VDH, TDH, EB, and CB are 43.1 mm, 42.8 mm, 39.6 mm, 56.9 mm, and 39.9 mm, respectively.

**Table 2:** Demarking point for males and females

Variable	Demarking point	Wilks’ lambda	p-value
MLH	F< 290.5	0.516	<0.001
MDH	F< 43.1	0.548	<0.001
VDH	F< 42.8	0.494	<0.001
TDH	F< 39.6	0.676	<0.001
EB	F< 56.9	0.631	<0.001
CB	F< 39.9	0.725	<0.001

The accuracy of measurements of the humerus in determining sex is represented in Table 3. The accuracy of MLH was 81% in males, 94% in females, and 85% in the total population. The accuracy of MDH, VDH, and TDH was 69%, 90%, and 75% in males; 92%, 75%, and 77% in females; and 76%, 85%, and 76% in the total population, respectively. EB and CB correctly identified 78% and 81% of male bones, 67% and 71% of female bones, and 75% and 78% of all bones.

**Table 3:** Percentage of correctly classified cases

Variable	Male	Female	Average
MLH	81%	94%	85%
MDH	69%	92%	76%
VDH	90%	75%	85%
TDH	75%	77%	76%
EB	78%	67%	75%
CB	81%	71%	78%

## Discussion

Measurements of long bones have significant medico-legal importance in the identification of missing persons. The humerus is the chief bone of the upper limb and it maintains its integrity long after the body has decomposed. The length of the humerus has been reported to be important in the identification of specific features of the population [20]. In our study, the average length of the humerus was 304.56 ± 14.16 mm and 276.60 ± 10.89 mm in males and females, respectively. In Brazil, a study was conducted and it found the average humerus length to be 31.3 ± 2.3 [21] cm while MLH was found to be 30.28 ± 2.44 cm in a study conducted in southern India [22]. A profound difference was observed by Devi et al. [23] between the length of the right and left humerus of the same body with the average right-sided length being 30.84 ± 1.78 cm. MLH was 30.78 ± 1.57 cm in a study conducted on the northern Thai population [24].

Segmental measurements of the humerus can be used to estimate the length of the humerus. Gayatri et al. [25] observed a significant relationship of epicondylar breadth and the vertical diameter of the head of the humerus with the maximum length of the humerus. Similar results were observed in the study conducted by Udhaya et al. [22]. A significant relationship of segmental measurements with the length of the humerus was also observed by Salles et al. [26].

For a forensic expert, the determination of gender is the first step in the identification of an unknown person. It is common practice to use a discriminant function equation to determine gender from the skeletal remains of the individual. But it has been understood that these equations are population specific. This has made it compulsory to develop separate equations for every regional population [27]. We observed in our study that there is a noteworthy difference in all humeral measurements between the male and female groups, which shows sexual dimorphism in all the segmental measurements of the humerus in the South-East Asian population. The discriminant functions derived in our study will be of substantial help for medico-legal specialists in the region.

In our study, we observed that maximum humeral length and VDH are good dimensions for the differentiation of sex with correct sexual differentiation being 85%. Lee et al. [19] publicized that VDH had the highest accuracy for the differentiation of sex. Patil et al. [28] conducted a study in southern India and observed that the length of the humerus and the mid-shaft diameter of the humerus are good measurements for gender identification.

The single finest factor for gender determination was epicondylar breadth (with 87.5% accuracy) and was observed by Soni et al. [29] while vertical diameter was the best parameter in another study [7]. These differences in measurement are expected to be dependent on the difference in the body size, muscle mass, and physical activity, significantly more development of the cortical bone in adolescent males than in females, and differences in bone remodeling between both genders [30].

## Conclusions

This study shows that the maximum humeral length and the vertical diameter of the humeral head are the two most important measurements with the highest accuracy for the determination of gender from the skeletal remains of the human body in the South-East Asian population.
